# Effects of a Telenutrition Weight Loss Program Supported with Telemonitoring and Telehealth Coaching on Anthropometric and Biochemical Measures in Overweight and Obese Adults: A Pilot Randomized Controlled Trial

**DOI:** 10.3390/healthcare12232362

**Published:** 2024-11-25

**Authors:** Noura M. S. Eid, Ebtisam A. Al-Ofi, Sumia Enani, Rana H. Mosli, Raneem R. Saqr, Karimah M. Qutah, Sara M. S. Eid

**Affiliations:** 1 Department of Clinical Nutrition, Faculty of Applied Medical Sciences, King Abdulaziz University, Jeddah 21589, Saudi Arabia; aimuwseli@kau.edu.sa; 2 King Fahd Medical Research Center, King Abdulaziz University, Jeddah 21589, Saudi Arabia; ealofi@kau.edu.sa (E.A.A.-O.); senani@kau.edu.sa (S.E.); eidsar@gmail.com (S.M.S.E.); 3 Department of Physiology, Faculty of Medicine, King Abdulaziz University, Jeddah 21589, Saudi Arabia; 4 Department of Food and Nutrition, Faculty of Human Sciences and Design, King Abdulaziz University, Jeddah 21589, Saudi Arabia; 5 Department of Management Information System, Faculty of Economics and Administration, King Abdulaziz University, Jeddah 21589, Saudi Arabia; rsaqr@kau.edu.sa; 6 The Management of Digital Transformation and Innovation Systems in Organization Research Group, King Abdulaziz University, Jeddah 21589, Saudi Arabia; 7 Horizon Health Network, Fredericton, NB E3B 5N5, Canada; kqutah@gmail.com

**Keywords:** obesity, weight loss, telenutrition, telemonitoring, telehealth coaching

## Abstract

Background/Objectives: Telenutrition has been reported to enable dietitians to support outpatients remotely in an accessible and convenient state. The literature shows that both telemonitoring and health coaching are effective approaches for achieving a greater impact on weight loss and the client’s compliance with their diet. The purpose of the present study was to assess the impact of a telenutrition weight loss program on weight reduction, supported with telemonitoring and health coaching. Methods: A pilot randomized controlled trial was conducted among overweight and obese adults. The control group (n = 11) received a hypocaloric and tailored diet via telenutrition. The intervention group (n = 18) also received a hypocaloric and tailored diet via telenutrition, but it was supported with weekly telemonitoring and monthly telehealth coaching. Results: Participants who joined the intervention group showed a reduction in weight, BMI, fat %, visceral fat, and WC, whereas participants in the control group only displayed a reduction in WC after a period of 3 months. Comparing between the groups, a greater reduction in weight, BMI, fat %, and visceral fat was seen in the intervention group. Furthermore, muscle % was significantly higher in the intervention group after a period of 3 months. At the 6-month follow-up, the intervention group still showed promising results, but they were not significant. Conclusions: Our data indicate that the integration of the three approaches of telenutrition, telemonitoring, and health coaching in dietary weight loss programs can have significant impacts on weight loss in obese and overweight individuals.

## 1. Introduction

The healthcare landscape is perpetually evolving, marked by a relentless pursuit of innovative solutions to address the multifaceted challenges encountered by individuals battling chronic conditions and improving their lifestyle via telemedicine [1]. Within this context, the convergence of telecare interventions and the management of chronic diseases, such as obesity and diabetes, has emerged as a focal point of exploration and advancement seen through telemonitoring [2]. However, dietetic practice has been shifting to some extent towards incorporating online consultations rather than traditional in-person consultations, as virtual care has been emerging as a promising strategy for promoting health and well-being in the dynamic landscape of today’s life [3]. In 2019, “Telenutrition” was defined by The Academy of Nutrition and Dietetics as “the interactive use by an RDN of electronic information and telecommunications technologies to implement the Nutrition Care Process with individuals at another location and following state laws” [4]. This modern approach not only addresses nutritional concerns but also impacts various facets of health, including weight management in obesity [5,6]. Obesity is defined by the World Health Organization (WHO) as a global epidemic chronic disease, resulting in more than 5 million deaths yearly around the world; the WHO identified combating this public crisis as one of the 2025 global nutrition targets (for children under five) and one of the Targets for Noncommunicable Disease (NCD) reduction for adolescents and adults [7]. Various treatments, such as dietary changes, physical activity, behavioral therapy, medications, surgery, or a combination of these approaches, can be accessible for both clinical and community groups via telenutrition [4]. However, challenges exist in their availability, costs, adherence to treatment, and their effectiveness over the long term [8]. The expense of these approaches is a significant issue within continuous care settings, posing obstacles to public health strategies [9]. Sustaining weight loss is the primary difficulty in addressing obesity, as most individuals regain around one-third of lost weight within a year [10]. Telenutrition is believed to provide better healthcare outcomes for patients than traditional care, and it reduces geographic barriers related to the management of health. Electronic devices used for tele-based monitoring include video conferencing, software, websites, email, mobile phones, and Bluetooth telecommunications resources [11,12]. Telehealth serves as a sustainable and cost-effective approach, reaching large populations of individuals with obesity. In the TECNOB study (Technology for Obesity), telehealth was thoroughly evaluated for its effectiveness in promoting adherence and the long-term adoption of healthy behaviors at minimal expense [5]. Additionally, telemonitoring has also been reported to support women with gestational diabetes mellitus in managing weight gain [13].

Previous studies have demonstrated the effectiveness of telenutrition in weight management in comparison with traditional dietetic consultations [6,14,15,16]. Other studies have shown that telemonitoring [2,13,17] and health coaching [18,19] also support patients in losing weight. Novel approaches are rapidly emerging and need further investigation to reach an ideal model of telenutrition that promotes sustainable weight loss and patient compliance for long-term healthy habits and lifestyle behaviors. We designed an integrative model for the first time by combining telenutrition, telemonitoring, and health coaching and compared it with a single-component telenutrition model to assess changes in weight and several health-related assessments [20]; such an integrative approach has not been considered in previous research. Additionally, we incorporated a novel health coaching approach, known as “Integrative Nutrition”, and utilized a newly validated assessment tool (Circle of Life (Col) Satisfaction Scale) [21] to monitor participants’ health status and provide a comprehensive view of their quality of life, thereby enhancing the support offered through health coaching. These components could fill gaps in the existing literature to provide a more comprehensive understanding of how an integrative approach may be more effective in achieving sustainable weight loss health improvements. Given the scarcity of telemedicine studies conducted in the Middle East, particularly in Saudi Arabia, this study has the unique opportunity to be the first of its kind to be implemented within the Saudi Arabian context. In the present study, we aimed to confirm the impact of telenutrition and expand our investigation to a comparison between the effect of home telenutrition combined with telemonitoring and personalized health coaching on weight loss vs. home telenutrition only. Furthermore, we assessed biochemical tests related to health and diet to evaluate the effectiveness of the intervention on health status.

## 2. Materials and Methods

### 2.1. Participants

This study’s detailed protocol is published in the *British Journal of Nutrition* [20]. Study participants consisted of adults of both genders, aged 20–50 years, who met the following criteria: (1) obese or overweight based on the World Health Organization (WHO) criteria of a BMI over or equal to 25 for overweight participants and a BMI over or equal to 30 kg/m2 for obese participants, and (2) informed and signed the consent form. Participants that required specific medical nutrition therapy were excluded, as were those who might have struggled using the online applications required for this study, such as WhatsApp and Zoom, and/or struggled with accessing the internet or downloading WhatsApp and Zoom on a compatible device. To achieve this, we excluded participants who were (1) not capable of understanding or not familiar with using the online applications required for this study; (2) pregnant or lactating women; (3) having or with a history of chronic diseases, such as diabetes or cardiovascular diseases, thyroid dysfunction, or any other endocrine abnormalities; (4) participation in any other weight loss programs or using medication to lose weight for the past 3 months; and (5) not being available for the whole 6-month trial duration. Sample size calculations using a significance level of 5%, power of 80%, and dropout rate of 25% indicated that, to detect a significant change in weight loss between the intervention (3.7 kg/2.5 SD) and control group (1.6 kg/2.5 SD), at least thirty-five participants were required [18]. A total of 70 patients were screened, and 50 participants were eligible for this study. This was followed by randomizing and dividing the participants into two groups. The sample size calculation was based on a published study that measured the effectiveness of telemedical coaching on weight loss [18], which was comparable to the current study’s outcomes. Our study aimed to highlight the primary outcomes of a pilot randomized study, so we only examined 10–30% of the targeted sample size based on sample size recommendations for pilot studies [22]. A pilot study was conducted to evaluate the newly developed “Integrative Model” approach within the selected population for the first time. To ensure a representative sample and account for potential attrition, a total of 50 participants were enrolled in this study, with 25 participants assigned to each group. Upon completion at the first 3 months, a total of 18 participants were retained in the control group and 11 in the intervention group, while upon completion at 6 months, a total of 9 participants were retained in the control group and 16 in the intervention group. Still, the attrition rate was lower in the intervention group than in the control group, where the reasons behind withdrawals from this study mainly related to external challenges and barriers, such as work, university, and home responsibilities [23].

### 2.2. Study Design

**Figure 1:**
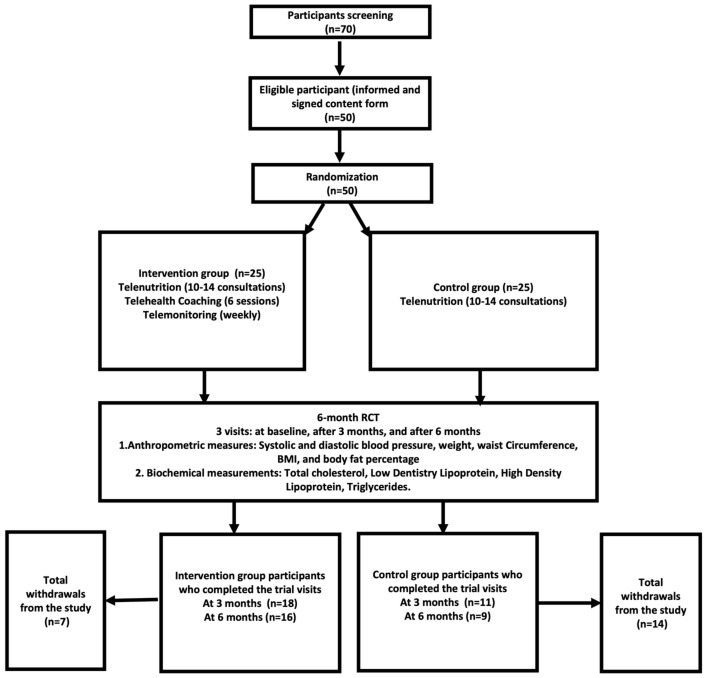
Participant flow diagram.

A pilot randomized control and intervention study was conducted in Jeddah, Saudi Arabia, between January 2022 and August 2023. The Research Ethics Committee (REC) at the Unit of Biomedical Ethics, Faculty of Medicine at King Abdul-Aziz University, approved the study protocol (HA-02-j-008). This study was designed as a two-arm randomized controlled trial, where participants were randomly divided into two groups: the control group received a hypocaloric and tailored diet via home telenutrition by registered dietitians (RDs); the intervention group followed the integrative model, where they received a hypocaloric and tailored diet via home telenutrition by registered dietitians (RDs), in addition to support through weekly telemonitoring and monthly telehealth coaching by an integrative nutrition health coach. Additionally, participants in the intervention group were telemonitored weekly during the intervention period (total of 36 weeks). A participant flow diagram is depicted in Figure 1.

### 2.3. Program Description

Participants were recruited through the online platforms of the university and research center by sharing advertisement flyers that described the study purpose and contact details of the research assistants. The entire 6-month RCT was conducted in the Food, Nutrition and Lifestyle Unit at King Fahd Medical Research Center, Jeddah, Saudi Arabia. All recruited participants were invited to be screened for eligibility by a family doctor, followed by signing an informed consent form after eligibility was confirmed. All eligible participants were scheduled to attend three physical visits throughout the 6-month duration of the study to collect specific measurements at baseline, after 3 months, and after 6 months (anthropometric measurements and biochemical analysis). At the end of each visit, participants from both groups were invited to join an online educational talk about nutrition and health. Regarding the intervention group, each participant received a bag containing “self-recording devices”, such as a weight scale, a blood pressure analyzer, and an armband measuring daily steps to be recorded during the weekly telemonitoring sessions. Telemonitoring was carried out via WhatsApp messages by an integrative nutrition health coach to gather the following health information: body weight, blood pressure, steps, and quality of life information [13]. In addition, at the end of each month, participants in the intervention group were invited to join one-on-one and/or group telehealth coaching sessions delivered by an integrative nutrition health coach to use the information gathered via telemonitoring and support the participants on their journey towards better health, considering spirituality, creativity, finances, career, education, health, physical activity, home cooking, home environment, relationships, social life, and joy [24]. The aim of health coaching was to tackle the factors associated with their eating habits and behavior to support weight loss. The research assistants ensured that all participants downloaded the WhatsApp business application (Version 2.24.23.79 for Android devices) and Zoom conference call software (Version 6.2.10 for Windows, macOS, and Linux platforms.) for the purposes of telemonitoring and health coaching.

### 2.4. Anthropometric Measurements and Blood Biochemical Measures

Anthropometric measurements, including systolic and diastolic blood pressure, weight, waist, and BMI, and body composition analyzer measurements, including fat percentage, muscle percentage, and visceral fat percentage, were recorded during each visit using standardized equipment and techniques. Samples were collected at the Food, Nutrition and Lifestyle unit and sent to ROYA First Vision Laboratories in King Fahd Center for Medical Research to carry out the following tests: total cholesterol, low-dentistry lipoprotein, high-density lipoprotein, and triglycerides. The obtained results were determined to be appropriate according to the specific range of quality control values given by the manufacturers (https://royakau.com/en/ (accessed on 1 January 2022).

### 2.5. Statistical Analysis

Data were analyzed using SPSS program version 26.0. Continuous variables were reported as median and interquartile range (IQR) or mean and standard deviation (SD) where appropriate, while categorical variables were reported as frequencies and percentages (%). The analyses were conducted separately for all-time-point completers and any-time-point completers.

For all-time-point completers, within-group comparisons (baseline vs. 3 months and baseline vs. 6 months) were analyzed using related-samples Friedman’s two-way analysis of variance by ranks. Pairwise comparisons for within-group changes were adjusted using the Bonferroni correction for multiple tests. Between-group comparisons (intervention vs. control) at each time point were performed using the Mann–Whitney U test, given the small sample size.

For any-time-point completers, the Mann–Whitney U test was used to evaluate the median between the intervention and control groups.

For categorical variables, between-group differences were examined using the Chi-square test. For all analyses, a two-sided *p*-value of less than 0.05 was considered statistically significant.

## 3. Results

### 3.1. Participants’ Characteristics

A total of 29 participants (18 and 11 in the intervention and control arms, respectively) completed the 3-month visit, and a total of 25 participants (16 and 9 in the intervention and control arms, respectively) completed the 6-month visit. Both arms were balanced as the baseline characteristics of participants were not significantly different between the two study groups, as shown in Table 1.

### 3.2. Changes in Anthropometric and Body Composition Measurements from Baseline to 3 and 6 Months

Changes in anthropometric and body composition measurements from baseline to 3 and 6 months for all-time-point and any-time-point completers are shown in Table 2 and Table 3, respectively.

For all-time-point completers, significant within-group changes were observed in the intervention group at the 3-month, but not the 6-month, follow-up period (Table 2). At 3 months, the intervention group experienced a median weight reduction of −3.65 kg (IQR: −5.55 to −0.95) compared to the control group’s minimal reduction of −0.2 kg (IQR: −1.73 to 0.8), with a statistically significant difference between groups (*p* = 0.012; Table 2). The BMI was reduced from a median of 32.5 (IQR: 28–37) to 29.3 (IQR: 27–34, *p* < 0.01; Table 2) in the intervention group only. Additionally, the fat percentage significantly declined in the intervention group from 44.9% (IQR: 38–49) to 40.7% (IQR: 36–48, *p* < 0.01; Table 2), and the muscle percentage showed an increase from a median of 24.9% (IQR: 22–28) to 25.7% (IQR: 23–31, *p* < 0.05; Table 2). At 6 months, the intervention group continued to maintain reductions in weight, BMI, and fat percentage compared to baseline, but these changes did not reach statistical significance.

In the between-group analysis, the intervention group showed significantly greater improvements than the control group at 3 months for weight, BMI, and fat percentage. The median weight change in the intervention group was −3.65 kg (IQR: −5.55 to −0.95), compared to −0.2 kg (IQR: −1.73 to 0.8) in the control group (*p* = 0.012; Table 3). Similarly, the BMI decreased by −1.35 (IQR: −2.38 to −0.38) in the intervention group versus −0.05 (IQR: −0.68 to 0.2) in the control group (*p* = 0.019; Table 3), and the fat percentage was reduced by −2.55% (IQR: −4.78 to −0.75) in the intervention group, compared to −0.9% (IQR: −2.35 to 0.13) in the control group (*p* = 0.044; Table 3). The muscle percentage change did not reach statistical significance between groups. By 6 months, the differences in weight, BMI, and fat percentage between the groups were no longer statistically significant. Waist circumference (WC) changes did not show significant differences at either time point, although both groups experienced reductions.

The three-month weight change, stratified by the BMI level and gender, demonstrated significant differences between the intervention and control groups in certain subgroups (Table 4). For participants with a BMI ≥ 30, the intervention group had a median weight reduction of −4.8 kg (IQR: −9 to −1.4), compared to a median increase of 0.7 kg (IQR: −1.15 to 1.75) in the control group, with a statistically significant difference (*p* = 0.012; Table 4). Among male participants, those in the intervention group lost a median of −5.8 kg (IQR: −11 to −4.5), which was significantly greater than the −0.4 kg (IQR: −2.1 to 0.8) median weight change in the control group (*p* = 0.009; Table 4). In contrast, female participants in the intervention group had a median weight reduction of −2.9 kg (IQR: −4.2 to −0.8), while those in the control group had a slight increase of 0.6 kg (IQR: −0.2 to 0.8), though this difference did not reach statistical significance. For participants with a BMI < 30, similar patterns in weight changes were observed, with no statistically significant difference between the intervention and control groups.

### 3.3. Clinical and Biochemical Measurements at All Time Points

For all-time-point completers, the within-group analysis showed that the intervention group showed significant reductions in systolic blood pressure, cholesterol, HDL, LDL, and triglycerides at 3 months. Specifically, the systolic blood pressure decreased from 126 mmHg (IQR: 118–141) at baseline to 117 mmHg (IQR: 110–125, *p* < 0.05; Table 5) at 3 months. The cholesterol levels dropped from 5.13 mmol/L (IQR: 4.67–5.77) to 4.64 mmol/L (IQR: 4.16–5.1, *p* < 0.05; Table 5), and the HDL levels decreased from 1.36 mmol/L (IQR: 1.15–1.45) to 1.07 mmol/L (IQR: 1.04–1.22, *p* < 0.01; Table 5). The LDL levels also significantly declined, from 4.69 mmol/L (IQR: 4.1–4.97) to 3.4 mmol/L (IQR: 3–4, *p* < 0.001; Table 5). At 6 months, the intervention group maintained some trends in these measurements, though without further statistically significant changes from baseline. In contrast, the control group showed no significant improvements within group over the same period.

In the between-group analysis for any-time-point completers, the LDL levels demonstrated a significant between-group difference by 6 months, with the intervention group showing a lower median LDL (3.57 mmol/L, IQR: 3.17–4.04) compared to the control (4.08 mmol/L, IQR: 3.83–4.44, *p* = 0.04; Table 5). The TG levels were also significantly lower in the intervention group at 3 months (median: 0.93 mmol/L, IQR: 0.73–1.1) compared to the control group (median: 1.21 mmol/L, IQR: 0.91–1.44), with a statistically significant difference (*p* = 0.035; Table 5). At 6 months, the intervention group continued to show lower triglyceride levels (median: 0.85 mmol/L, IQR: 0.59–1.04) compared to the control (median: 1.62 mmol/L, IQR: 1.23–1.79), with this difference approaching statistical significance (*p* = 0.05; Table 5). Other markers, such as systolic and diastolic blood pressure levels, showed favorable trends but no significant between-group differences.

## 4. Discussion

Home telemonitoring has shown effective results in patients with various chronic illnesses, such as diabetes and hypertension [25,26,27,28], and it improves outcomes in the management of obstetrics cases and complicated pregnancies [29]. The present study provides evidence that a telemonitoring approach may be effective for obesity management and weight reduction. At 3 months of the intervention, participants in the present study who were receiving both telemonitoring and health coaching experienced a reduction in weight, BMI, fat %, visceral fat, and WC. These participants also showed an increase in muscle % after following the intervention for 3 months. On the other hand, participants in the control group only exhibited a reduction in WC after a period of 3 months. Comparing the intervention group to the control group, the decrease in weight, BMI, fat %, and visceral fat was significantly greater. Furthermore, participants in the intervention group showed a greater increase in muscle % after a period of 3 months compared to participants in the control group. At the 6-month follow-up, although participants in the intervention group also showed a greater reduction in weight, BMI, fat %, visceral fat, and WC, as well as a greater increase in muscle %, compared to participants in the control group, none of these differences were statistically significant. In agreement with our current findings, previous research that implemented telemedical coaching for weight management showed that it contributed to significant weight loss in overweight employees [18]. However, our findings provide a detailed analysis of body weight composition, which was altered significantly among participants in the intervention group. In addition, a retrospective study was conducted to examine the impact of health coaching on both weight loss and weight loss percentages, revealing a strong association between attending five or more health coaching sessions and achieving at least a 5% reduction in body weight [30]. In accordance with previous findings [31], the analysis of our biochemical data showed that the telemonitoring and health coaching approach had a positive effect on the blood lipid profile; participants in the intervention group had a significantly lower blood triglyceride level compared to participants in the control group at the 3-month follow-up. Additionally, participants in the intervention group had significantly lower triglyceride and LDL levels at the 6-month follow-up compared to participants in the control group. A comprehensive literature review examining a large number of RCTs confirmed that both telemonitoring and health coaching exhibit a strong influence on weight loss outcomes [32]. The findings underline the potential of integrative approaches for sustainable, accessible, and scalable weight management, especially for those at risk of obesity-related health issues.

One of the main roles of health coaches is to follow up with telemonitoring data, which has an impact on clients’ health and well-being, such as stress [33], sleep [34], and physical inactivity [35]. In the current study, participants were coached to set specific goals to support their commitment to the hypocaloric diet, which is defined as “integrative nutrition”. A previous study, conducted among patients with obstructive sleep apnea in Japan, found that enhancing continuous positive airway pressure (CPAP) telemonitoring with electronic scales, BP monitors, and pedometers led to greater body weight reductions [36]. On the contrary, a randomized controlled trial involving pregnant females found that a self-directed, telemonitoring intervention did not result in less excessive weight gain during pregnancy [37]. Although there are conflicting findings regarding the effectiveness of telemonitoring and internet-based interventions on weight reduction [37,38], the discrepancies may be due to the components of each intervention and whether or not it includes coaching sessions or personalized counseling with professionals. The present study included one-on-one online coaching sessions delivered by an integrative nutrition health coach to reflect on the monitored weekly health measurements, which was found to be effective when combined with telemonitoring in the intervention group in improving weight and body composition outcomes among obese patients. This is in line with a previous study, which also incorporated weekly coaching sessions into its telemonitoring intervention and found this approach to result in greater weight loss among overweight employees [18]. The diminished significance in differences between the two groups might be explained by a lack of adherence to lifestyle changes among the participants, as previously observed at 6 months during weight loss programs [39]. Additionally, metabolic adaptations that occur following a period of weight reduction may have contributed to the plateau in weight loss at 6 months [40]. According to the results from our stratified analysis, participants who had a BMI ≥ 30 kg/m^2^ lost more weight when supported with telemonitoring and health coaching compared to participants who had a BMI < 30 kg/m^2^. Previous research also showed that higher BMI categories were associated with a greater reduction in body weight [38]. The higher weight loss among obese individuals compared to overweight individuals might be due to the higher energy expenditure [41]. Additionally, our findings showed that the telemonitoring and health coaching yielded greater weight loss among men compared to women, which is consistent with findings from previous weight loss interventions [42]. This difference between men and women might be explained by the possibility that only highly motivated men participated in this study, as the majority of participants were women (62.1%), which is similar to what has been observed in previous research [43].

Our findings, therefore, highlight the importance of considering both BMI and gender when evaluating the impact of weight loss interventions, indicating that telemonitoring could offer a tailored approach to support significant weight loss in more obese individuals, particularly males. Indeed, the intervention group engaged in a holistic, integrative nutrition approach to weight management, which likely led to better outcomes and contributed to a lower attrition rate compared to the control group, indicating the intervention’s positive impact. One common factor leading to dropout in the weight loss study is difficulty with the weight loss itself, which is often associated with age as well as psychological and behavioral factors [44,45]. As mentioned earlier, the Institute for Integrative Nutrition’s “circle of life” model was used to assess the participants’ CoL Satisfaction Scale results [21] regarding several lifestyle components, which had a strong impact on the participants’ commitment and motivation to remain in the weight loss program.

To our knowledge, the present study is the first in Saudi Arabia to assess the effectiveness of a telemonitoring program that incorporated personalized nutritional counseling and health coaching for weight management among overweight and obese individuals. A key strength of this study is its multi-component design, allowing for a comprehensive assessment of the combined effects of telenutrition, telemonitoring, and telehealth coaching on weight and anthropometric measurements. Additionally, the research team included a multidisciplinary group of health professionals, including a family medicine physician, a clinical dietitian, and integrative nutrition health coaches, to support participants’ health and weight management. This collaboration underlines the value of integrative approaches in achieving comprehensive care. On the other hand, this study has several limitations. First, a three-arm design, comparing each approach, telenutrition, telemonitoring, and health coaching, were not implemented, which may have weakened the reliability of the results, as it is not known whether the obtained outcomes were due to telemonitoring or telehealth coaching. Furthermore, the baseline and follow-up assessments, as well as nutrition counseling sessions, were conducted by the same research group members, and blinding was, therefore, not feasible. Additionally, as a pilot study, the sample size was limited, suggesting the need for a larger sample size and a longer trial period (ideally 12 months) in future research. In addition, the dropout rate was high, potentially weakening this study’s strengths and influencing the reliability of the results. The current study was a pilot randomized study that implemented the novel “Integrative Model” approach for the first time with a limited sample size (10–30% of the target), as suggested in the literature. A total of 50 participants were enrolled, evenly divided into two groups, to ensure representation while accounting for potential attrition [22].

## 5. Conclusions

The current study was preliminary in nature, demonstrating a personalized approach for weight management programs. The data confirmed significant reductions in BMI, fat %, visceral fat, and WC and an increase in muscle % among participants in the intervention group in comparison with the control group. Future research should be directed towards exploring the use of established telemedicine platforms and/or developing a new telemedicine platform in Saudi Arabia, which is currently not available in the country. Supporting participants’ self-recording with telemonitoring devices would have strengthened the accuracy and technological foundation of this study. For future studies, using a validated WHO quality of life (QOL) tool is necessary to provide a more comprehensive understanding of each intervention’s specific impact. The WHO QOL measure captures the following four domains: physical, psychological, social, and environmental. Further, an end-of-project evaluation survey is essential to examine the participants’ commitment, motivation, enjoyment level, and challenges after being monitored weekly and coached by integrative nutrition health coaches. Assessing motivation and readiness to change prior to the intervention might be an important step for interpreting findings in future studies. We also suggest monitoring participants’ adherence to dietary changes via telemonitoring to confirm significant improvements in eating patterns and lifestyle. Thus, in our future research, we aim to conduct an actual RCT with the total target sample size following our published study protocol [20] and for a longer intervention period (12 months), where the targets may be achieved when set for a longer period of time [46]; for the participants in the current study, the duration of 3 and 6 months might not have been long enough to reflect sustainable changes in weight and body composition.

## Figures and Tables

**Table 1:** Baseline characteristics of participants.

	Intervention (n = 18)	Control (n = 11)	*p*-Value
Age (years) (Mean ± SD)	33 ± 11	36 ± 10	0.469
Gender (n, %)			
Men	5, 28%	6, 54.5%	0.523
Women	13, 72%	5, 45.5%	
Weight (kg) (Mean ± SD)	90.1 ± 20.7	90.9 ± 21.9	0.922
BMI (Mean ± SD)	33.2 ± 6.15	33.9 ± 5.58	0.744
BMI (n, %)			
<30	7, 39%	3, 27%	0.159
≥30	11, 61%	8, 73%	
Fat % (Mean ± SD)	45 ± 7.95	42.4 ± 9.1	0.419
Muscle % (Mean ± SD)	24.6 ± 4.53	25.9 ± 4.79	0.485
Visceral fat % (Mean ± SD)	10.8 ± 5.01	13.1 ± 5.96	0.283
WC (CM) (Mean ± SD)	97 ± 16	102 ± 18	0.352

**Table 2:** Changes in weight, BMI, body composition, and waist circumference over time for all-time-point completers.

	Baseline Median (IQR)	3 Months ^a^ Median (IQR)	6 Months ^a^ Median (IQR)	Difference at 3 Months from Baseline ^b^	Difference at 6 Months from Baseline ^b^
Weight					
Intervention	82.7 (74 to 108)	79.4 (73 to 97) **	77.9 (70 to 100)	−3.65 (−5.55 to −0.95) ^	−2.9 (−6.6 to −0.25)
Control	86.7 (74 to 116)	85.5 (72 to 117)	85.6 (72 to 113)	−0.2 (−1.73 to 0.8)	−0.5 (−3.43 to 0.55)
BMI					
Intervention	32.5 (28 to 37)	29.3 (27 to 34) **	30.3 (27 to 34)	−1.35 (−2.38 to −0.38)	−1.15 (−2.58 to −0.18)
Control	35.1 (29 to 38)	34.8 (28 to 39)	34.6 (28 to 39)	−0.05 (−0.68 to 0.2)	−0.25 (−1.23 to 0.1)
Fat %					
Intervention	44.9 (38 to 49)	40.7 (36 to 48) **	44.3 (37 to 48)	−2.55 (−4.78 to −0.75)	−0.7 (−3.65 to 0.45)
Control	42.1 (36 to 50)	40.7 (34 to 48)	41.6 (36 to 47)	−0.9 (−2.35 to 0.13)	−0.3 (−1.68 to 0.68)
Muscle %					
Intervention	24.9 (22 to 28)	25.7 (23 to 31) *	24.4 (21 to 30)	1.9 (0.13 to 2.75)	0.2 (−0.2 to 2.1)
Control	25.9 (22 to 30)	26.7 (22 to 31)	26.1 (23 to 30)	0.55 (0.1 to 1.15)	0.2 (−0.3 to 1.25)
Visceral fat %					
Intervention	11 (6.8 to 13.5)	9.5 (6.8 to 11.3)	9.5 (6 to 12)	−0.5 (−2 to 0)	−0.25 (−1 to 0)
Control	14 (8.8 to 20)	14 (8 to 21)	14 (7.8 to 20)	0 (−0.75 to 0.75)	0 (−1 to 0)
WC					
Intervention	92.3 (84 to 111)	87 (79 to 98) *	86.8 (79 to 105)	−4.5 (−8.13 to −2)	−4.25 (−8.5 to 0)
Control	104 (92 to 123)	103 (86 to 120)	93.5 (85 to 124)	−3.5 (−7.5 to −0.75)	−5 (−8.75 to 0.75)

Values are presented as median (IQR). ^a^ *p*-values are based on related-samples Friedman’s two-way analysis of variance by ranks, with pairwise comparisons adjusted using the Bonferroni correction for multiple tests. Within-group analyses compared values at baseline with values at 3 months and 6 months for each group. The analysis included all-time-point completers only (n = 16 for the intervention group and n = 8 for the control group) (*p*-values: * *p* < 0.05 and ** *p* < 0.01). ^b^ *p*-values are based on Mann–Whitney test for between-group (intervention vs. control) comparisons (*p*-values: ^ *p* < 0.05).

**Table 3:** Comparison of changes in anthropometric measurements between intervention and control groups at 3 and 6 months for any-time-point completers (intention-to-treat analysis).

Variable/Timepoint	Intervention		Control		*p*-Value
	n	Median (IQR)	n	Median (IQR)	
Weight (kg)					
Change at 3 Months	18	−3.65 (−5.8 to −1) *	11	−0.2 (−2.1 to 0.8)	0.012
Percentage Change		−3.91 (−5.78 to −1.14) *		−0.17 (−2.22 to 1.13)	0.016
Change at 6 Months	16	−2.9 (−6.5 to −0.6)	9	0.2 (−2.6 to 0.6)	0.108
Percentage Change		−3.17 (−7.58 to −0.83)		0.27 (−2.75 to 0.85)	0.108
BMI					
Change at 3 Months	18	−1.35 (−2.2 to −0.3) *	11	0 (−0.8 to 0.2)	0.019
Percentage Change		−4.15 (−6.59 to −1.15) *		0 (−2.31 to 0.56)	0.019
Change at 6 Months	16	−1.15 (−2.55 to −0.35)	9	−0.1 (−1 to 0.1)	0.136
Percentage Change		−4.01 (−7.95 to −1.04)		−0.39 (−2.88 to 0.36)	0.136
Fat %					
Change at 3 Months	18	−2.55 (−4.7 to −0.9) *	11	−0.6 (−2.4 to 0.2)	0.044
Percentage Change		−5.8 (−10.07 to −1.96)		−1.33 (−5.47 to 0.46)	0.071
Change at 6 Months	16	−0.7 (−3.5 to 0.4)	9	0.3 (−1 to 0.7)	0.329
Percentage Change		−1.35 (−8.11 to 1)		0.67 (−3.57 to 2.02)	0.411
Muscle %					
Change at 3 Months	18	1.6 (0.35 to 2.7)	11	0.5 (0 to 1.3)	0.089
Percentage Change		7.28 (1.59 to 10.92) *		2.32 (0 to 4.94)	0.033
Change at 6 Months	16	0.2 (−0.2 to 2.1)	9	−0.2 (−0.3 to 0.8)	0.29
Percentage Change		0.6 (−0.78 to 8.14)		−0.66 (−1.18 to 2.52)	0.929
Visceral Fat %					
Change at 3 Months	18	0 (−1.5 to 0)	11	0 (−1 to 0)	0.148
Percentage Change		0 (−15.34 to 0)		0 (−4.17 to 0)	0.148
Change at 6 Months	16	0 (−1 to 0)	9	0 (−1 to 0)	0.558
Percentage Change		0 (−10 to 0)		0 (−4.17 to 0)	0.599
WC (CM)					
Change at 3 Months	18	−5.25 (−8.5 to −2)	11	−3 (−6 to −3)	0.363
Percentage Change		−5.57 (−8.33 to −2.53)		−3.8 (−5.04 to −2.42)	0.220
Change at 6 Months	16	−4.25 (−8 to 0)	9	−3 (−8 to 1)	0.846
Percentage Change		−4.02 (−8.08 to 0)		−3.26 (−7.53 to 0.87)	0.803

Data are presented as median (IQR) for both the actual change in measurement and the percentage change from baseline. The analysis included any-time-point completers, following an intention-to-treat approach. *p*-values indicate the significance of between-group differences based on the Mann–Whitney U test * *p* < 0.05.

**Table 4:** Three-month weight change (kg) in each arm stratified by BMI level and gender.

	Intervention n = 18	Control n = 11	*p*-Value
BMI			
<30 (34.5%)	−2.1 (−4.2 to 0.3)	−0.6 (−3 to −0.2)	0.833
≥30 (65.5%)	−4.8 (−9 to −1.4) *	0.7 (−1.15 to 1.75)	0.012
Gender			
Male (37.9%)	−5.8 (−11 to −4.5) **	−0.4 (−2.1 to 0.8)	0.009
Female (62.1%)	−2.9 (−4.2 to −0.8)	0.6 (−0.2 to 0.8)	0.173

Data are presented as median (IQR) for both the actual change in measurement and the percentage change from baseline. The analysis included any time-point completers, following an intention-to-treat approach. *p*-values indicate the significance of between-group differences based on the Mann–Whitney U test * *p* < 0.05 and ** *p* < 0.01.

**Table 5:** Clinical and biochemical measurements at all time points.

	All-Time-Point Completers ^a^		Any-Time-Point Completers ^b^				
Variable/Timepoint	Intervention (n = 16)	Control (n = 8)	Intervention		Control		*p*-Value
	Median (IQR)	Median (IQR)	n	Median (IQR)	n	Median (IQR)	
Systolic BP							
Baseline	126 (118–141)	136 (118.5–150)	18	130.5 (119–146)	11	128 (124–147)	0.74
3 months	117 (110–125) *	123.5 (116–126)	18	117.5 (110–123)	11	122 (111–125)	0.363
6 months	126 (116–138)	127 (117–134.5)	16	126 (115.5–137.5)	9	122.5 (117–133)	0.881
Diastolic BP							
Baseline	73 (70–88)	85.5 (81.5–93)	18	75.5 (70–89) ^	11	87 (82–94)	0.024
3 months	73.5 (71–78.5)	76.5 (74–85.5) *	18	74 (72–79)	11	78 (73–85)	0.317
6 months	81 (72.5–88)	83 (77.5–86)	16	81 (72.5–88)	9	81 (77.5–86)	0.976
Cholesterol (mmol/L)							
Baseline	5.13 (4.67–5.77)	5.58 (5.01–6.43)	18	5.36 (4.79–5.92)	11	5.18 (5.03–6.11)	0.363
3 months	4.64 (4.16–5.1) *	4.72 (4.33–6.04)	18	4.8 (4.34–5.16)	11	4.9 (4.52–5.6)	0.387
6 months	5.09 (4.44–5.36)	5.52 (5.16–5.7)	16	5.09 (4.44–5.36)	9	5.53 (5.33–5.83)	0.059
HDL (mmol/L)							
Baseline	1.36 (1.15–1.45)	1.27 (1.19–1.43)	18	1.36 (1.14–1.45)	11	1.3 (1.19–1.52)	0.912
3 months	1.07 (1.04–1.22) **	1.06 (0.9–1.16) *	18	1.07 (1.03–1.26)	11	1.09 (0.9–1.23)	0.611
6 months	1.06 (1.01–1.2)	1.02 (0.87–1.27)	16	1.06 (1.01–1.2)	9	1.02 (0.9–1.23)	0.392
LDL (mmol/L)							
Baseline	4.69 (4.1–4.97)	4.79 (3.8–5.82)	18	4.74 (4.1–5.18)	11	4.56 (3.91–5.22)	0.912
3 months	3.4 (3–4) ***	3.84 (3.11–4.99)	18	3.47 (3.21–4.04)	11	3.66 (2.9–4.66)	0.387
6 months	3.57 (3.17–4.04)	4.04 (3.7–4.38)	16	3.57 (3.17–4.04) ^	9	4.08 (3.83–4.44)	0.04
TG (mmol/L)							
Baseline	0.9 (0.62–1.39)	1.1 (0.9–1.15)	18	0.9 (0.63–1.52)	11	1.12 (0.91–1.58)	0.204
3 months	0.87 (0.72–1.05)	1.28 (1.1–1.4) *	18	0.93 (0.73–1.1) ^	11	1.21 (0.91–1.44)	0.035
6 months	0.85 (0.59–1.04)	1.47 (1.15–1.8)	16	0.85 (0.59–1.04)	9	1.62 (1.23–1.79)	0.05

Values are presented as median (IQR). ^a^ *p*-values are based on related-samples Friedman’s two-way analysis of variance by ranks, with pairwise comparisons adjusted using the Bonferroni correction for multiple tests. Within-group analyses compared the baseline values with values at 3 months and 6 months for each group in all-time-point completers (*p*-values: * *p* < 0.05, ** *p* < 0.01 and *** *p* < 0.001). ^b^ *p*-values are based on Mann–Whitney test for between-group (intervention vs. control) comparisons. The analysis included any-time-point completers, following an intention-to-treat approach (*p*-values: ^ *p* < 0.05).

## Data Availability

The data presented in this study are available upon reasonable request from the corresponding author at ooaeid2@kau.edu.sa.
